# Preparation of Poly(acrylic acid-*co*-acrylamide)-*Grafted* Deproteinized Natural Rubber and Its Effect on the Properties of Natural Rubber/Silica Composites

**DOI:** 10.3390/polym14214602

**Published:** 2022-10-29

**Authors:** Supharat Inphonlek, Namthip Bureewong, Kasama Jarukumjorn, Pranee Chumsamrong, Chaiwat Ruksakulpiwat, Yupaporn Ruksakulpiwat

**Affiliations:** 1 School of Polymer Engineering, Institute of Engineering, Suranaree University of Technology, Nakhon Ratchasima 30000, Thailand; 2 Research Center for Biocomposite Materials for Medical Industry and Agricultural and Food Industry, Suranaree University of Technology, Nakhon Ratchasima 30000, Thailand

**Keywords:** modified natural rubber, graft copolymerization, poly(acrylic acid-*co*-acrylamide), natural rubber composites, mechanical property

## Abstract

This work aims to enhance the polarity of natural rubber by grafting copolymers onto deproteinized natural rubber (DPNR) to improve its compatibility with silica. Poly(acrylic acid-*co*-acrylamide)-*grafted* DPNR ((PAA-*co*-PAM)-DPNR) was successfully prepared by graft copolymerization with acrylic acid and acrylamide in the latex stage, as confirmed by FTIR. The optimum conditions to obtain the highest conversion, grafting efficiency, and grafting percentage were a reaction time of 360 min, a reaction temperature of 50 °C, and an initiator concentration of 1.0 phr. The monomer conversion, grafting efficiency, and grafting percentage were 91.9–94.1, 20.8–38.9, and 2.1–9.9%, respectively, depending on the monomer content. It was shown that the polarity of the natural rubber increased after grafting. The (PAA-*co*-PAM)-DPNR was then mixed with silica to prepare DPNR/silica composites. The presence of the (PAA-*co*-PAM)-DPNR and silica in the composites was found to improve the mechanical properties of the DPNR. The incorporation of 10 phr of silica into the (PAA-*co*-PAM)-DPNR with 10 phr monomer increased its tensile strength by 1.55 times when compared to 10 phr of silica loaded into the DPNR. The silica-filled (PAA-*co*-PAM)-DPNR provided s higher storage modulus, higher Tg, and a lower tan δ peak, indicating stronger modified DPNR/silica interactions and greater thermal stability when compared to silica-filled DPNR.

## 1. Introduction

Natural rubber (NR) has gained much interest as a biomaterial for various fields, such as the consumer industrial [1], automobile industrial [2], medical [3], and agricultural [4] sectors. NR is a biopolymer from rubber trees (*Hevea Brasiliensis*). Since it is a renewable and sustainable bio-based material, has high elasticity, and has the capability to form a film, it has the potential for use in various applications. Natural rubber composites have been developed in order to improve the mechanical properties of natural rubber, promoting efficiency for its application. Such improvements can be carried out by mixing the rubber with fillers, such as calcium carbonate [5], clay [6,7], and silica [8]. Silica, a compound of silicon and oxygen, is commonly found as quartz and sand in nature. It is an abundant mineral on the earth’s crust. It can be prepared from agricultural wastes, such as rice husk ash and sugar cane ash, by sol-gel and precipitation methods [9,10]. It has been applied as an adsorbent for removing contaminants [11] and an additive in the manufacturing industries [12]. Many research works have used silica as a reinforcing filler for rubber composites to enhance their properties, such as tensile strength, modulus, hardness, and abrasion resistance [13,14]. The silica-reinforced rubber composites were used for the fabrication of high-performance tires with wet grip ability and fuel saving efficiency [15]. According to the chemical structure of NR, it contains cis-1,4-polyisoprene, which is nonpolar. Therefore, it is incompatible with silica, resulting in the undesired properties of the products. This is one of the main drawbacks of the preparation of silica-based NR composites. Therefore, to overcome the incompatibility of silica with natural rubber, the polarity of natural rubber may be improved by introducing polar components.

Many attempts have been reported so far to enhance the polarity of NR and improve its compatibility with reinforcing fillers [16,17]. The modification of NR was accomplished by introducing polar functional components to its structure. Graft copolymerization is one of the most common methods for modification, in which the other polymer can be chemically bonded to the polymer backbone. From this method, the free radicals on the main chain are generated. The monomer is then added and undergoes polymerization to form a grafted copolymer. The graft copolymerization on the NR particles can be performed via surface functionalization in the latex stage, which allows for the additional functional groups on their particle surface. NR consists of polyisoprene chains and nonrubber components, including proteins, phospholipids, and fatty acids. The nonrubber components are in the aqueous medium and surface of the NR particles. The deproteinized natural rubber (DPNR) is employed in order to suppress the side reactions from the adsorbed proteins and can improve grafting efficiency [18]. J. Jayadevan et al. prepared DPNR grafted poly(dimethylaminoethyl methacrylate) (PDMAEMA) through emulsion polymerization to perform grafting modifications and followed this by blending it with poly(vinyl alcohol) (PVA) to fabricate membranes [19]. The polarity of the DPNR was increased after grafting, leading to a strong interfacial interaction with PVA. The enhancement of the mechanical properties was achieved by showing the increase in tensile strength and modulus. These membranes were used for the loading and releasing of model drugs, which could be interesting in biomedical fields. Therefore, it can introduce a variety of functional groups to natural rubber from this technique by the use of functional monomers. When increasing the polarity of natural rubber, the natural rubber should have additional functional groups, such as carboxylic acid, amine, amide, and hydroxyl. 

Acrylic acid, a carboxylic acid-containing monomer, can play an important role in improving the hydrophilicity of materials. Y. Cui et al. successfully prepared poly(acrylic acid)-*grafted* natural rubber (PAA-*g*-NR) via radical solution polymerization [20]. However, the organic solvent was used as a medium for this preparation system. These are the drawbacks in terms of cost and environmental considerations. The hydrophilicity of the resulting product increased after grafting with PAA. The grafted product was applied as a coating material for controlled release purposes. Moreover, the formed poly(acrylic acid) chains can also act as electrosteric stabilizers that improve colloidal stability for particles dispersed in an aqueous phase [21]. In addition, the copolymerization of acrylic acid and acrylamide has been studied to prepare efficient polyelectrolyte-based materials. The combination of polyacrylic acid and polyacrylamide provides a homogeneous structure with hydrophilic properties and hydrogen bonding formation, as well as enhancing its mechanical properties [22,23].

In this work, to improve the compatibility of natural rubber with silica, DPNR was modified by emulsion graft copolymerization with acrylic acid and acrylamide using an environmentally friendly process. The polymerization is carried out in latex stage, which is a water-based system. This system was safe as no organic solvents were used. The suitable condition for the preparation of the modified DPNR was optimized, and the physico-chemical properties of the modified DPNR were investigated. Specifically, the contact angle of the samples was examined in order to evaluate the polarity of the samples. The modified DPNR was then mixed with silica by a solid mixing process. The morphology, mechanical, and dynamic mechanical properties of the composites were also investigated.

## 2. Materials and Methods

### 2.1. Materials

Natural rubber latex (NR; 60% dry rubber content with high ammonia) was obtained from Chemical and Materials Co., Ltd. (Bangkok, Thailand). Sodium dodecyl sulfate (SDS) and acrylamide (AM) monomer were purchased from Loba Chemie Pvt. Ltd. (Mumbai, India). Acrylic acid (AA) monomer and cumene hydroperoxide (CHP) initiator were obtained from Aldrich (St. Louis, MO, USA). AA was purified by using a column packed with alumina adsorbent before polymerization [24]. Tetraethylene pentamine (TEPA) activator was purchased from Acros organics (Geel, Belgium). Urea and toluene were purchased from RCI Labscan Limited (Bangkok, Thailand). Terric16A (10 wt%) and 1,3-diphenyl guanidine (DPG) were obtained from the Rubber Authority of Thailand (Bangkok, Thailand). Ethanol was purchased from Duksan Reagents (Ansan, South Korea). The compounding ingredients, such as zinc oxide (ZnO), stearic acid, N-cyclohexyl-2-benzothiazole sulfenamide (CBS), and sulfur, were received from Chemical Innovation Co., Ltd. (Bangkok, Thailand). Precipitated silica (JS-185, surface area (BET) of 170–200 m^2^/g, a DBP absorption value of 2.0–2.6 cm^3^/g, and a residue from a 75 µm sieve ≤ 10%) was obtained from Jinsha Precipitated Silica Manufacturing Co., Ltd. (Fujian, China). Silica was used without further purification. Deionized water was used throughout the study.

### 2.2. Preparation of Poly(acrylic acid-co-acrylamide)-Grafted Deproteinized Natural Rubber ((PAA-co-PAM)-DPNR)

The deproteinized natural rubber was firstly prepared according to S. Kawahara et.al. [25] by incubation with 0.1 wt% of urea and 1 wt% of SDS for 60 min. The dispersion was centrifuged at 15,000 rpm for 30 min. The cream fraction was then redispersed with 1 wt% of SDS, followed by centrifugation. The washing step was performed two times. The cream fraction was collected, redispersed with deionized water, and kept for further modification. 

The modification of the DPNR by graft copolymerization with a comonomer of acrylic acid and acrylamide was carried out by the CHP/TEPA initiating system. The DPNR latex and Terric16A were charged into a glass reactor equipped with a mechanical stirrer. The latex was then purged with nitrogen gas for 45 min at a controlled temperature under stirring at 100 rpm. The CHP was charged into the reactor, followed by the addition of acrylic acid (40 mol% of acrylic acid was neutralized with 20 wt% of NaOH solution) and acrylamide. In this case, the comonomer ratio of the acrylic acid and acrylamide was kept constant at 50:50 by weight percentage. The monomer contents were varied as 10 and 30 phr, as shown in Table 1. Then, a TEPA solution was injected by dropwise addition to initiate polymerization. The weight ratio of CHP and TEPA was fixed at 1. The total solid content was kept constant at 20 wt%. Polymerization was continued in a nitrogen atmosphere under stirring. After that, the obtained latex was collected for characterization.

### 2.3. Characterization of (PAA-co-PAM)-DPNR

#### 2.3.1. Determination of Monomer Conversion

The prepared latex was poured into the plastic mold and dried at room temperature. The sample was further dried in a hot-air oven at 60 °C for 24 h. The sample sheet was weighed and immersed in ethanol for 24 h to remove the unreacted monomers. The extracted sheet was then dried at 60 °C for 24 h. The monomer conversion was determined by gravimetric analysis [26], as per the following equation.

(1) $$\mathrm{Monomer}\;\mathrm{conversion}\;\left(\%\right)=\frac{\mathrm{Weight}\;\mathrm{of}\;\left(\mathrm{PAA}-\mathrm{co}-\mathrm{PAM}\right)\;\mathrm{formed}}{\mathrm{Weight}\;\mathrm{of}\;\mathrm{monomer}\;\mathrm{charged}}\times 100.$$

#### 2.3.2. Determination of Grafting Efficiency and Grafting Percentage

The dried sample was extracted with DI water for 72 h to remove homo-(PAA-*co*-PAM). The water was changed three times a day. The samples were then dried in a hot-air oven at 60 °C for 24 h. The grafting efficiency and grafting percentage were calculated [27] as follows

(2) $$\mathrm{Grafting}\;\mathrm{efficiency}\;\left(\%\right)=\frac{\mathrm{Weight}\;\mathrm{of}\;\left(\mathrm{PAA}-\mathrm{co}-\mathrm{PAM}\right)-\mathrm{grafted}}{\mathrm{Weight}\;\mathrm{of}\;\mathrm{total}\;\mathrm{polymer}\;\mathrm{formed}}\times 100$$

(3) $$\mathrm{Grafting}\;\mathrm{percentage}\;\left(\%\right)=\frac{\mathrm{Weight}\;\mathrm{of}\;\left(\mathrm{PAA}-\mathrm{co}-\mathrm{PAM}\right)-\mathrm{grafted}}{\mathrm{Weight}\;\mathrm{of}\;\mathrm{DPNR}\;\mathrm{used}}\times 100.$$

#### 2.3.3. Fourier-Transform Infrared Spectroscopy 

The Fourier-transform infrared spectroscopy (FTIR) was used to study the chemical structure of the (PAA-*co*-PAM)-*grafted* DPNR using an attenuated total reflection (ATR) mode with a Tensor 27 FTIR spectrometer (Bruker, Billerica, MA, USA). The measurements were performed at a resolution of 4 cm^−1^ for 64 scans. The background was run before sample measurement. The FTIR spectra of all samples were recorded in the range of 4000–400 cm^−1^.

#### 2.3.4. Morphological Analysis

The morphology of the DPNR particles before and after modification was investigated using transmission electron microscopy (TEM). The dispersion was diluted and dropped onto a carbon-coated copper grid. The samples were stained with osmium tetroxide for 24 h [28]. The samples were subjected to a transmission electron microscope using Talos F200X (Thermo Fisher Scientific, Waltham, MA, USA) with a voltage of 120 kV.

#### 2.3.5. Particles Size Distribution 

The size and size distribution of the modified DPNR particles were determined by a laser-scattering particle size distribution analyzer using an LA-950V2 instrument (Horiba, Kyoto, Japan). The result was compared to the unmodified DPNR particles. The measurement was performed in the wet mode. The distilled water was used as a dispersant throughout the measurement. The samples were added into the chamber until the concentration was in the range of 1–5 wt% with ultrasonication during measurement. 

#### 2.3.6. Zeta Potential

The surface charge of the prepared latexes was measured by using Zetasizer 3000 (Malvern Panalytical Ltd., Worcestershire, UK). The samples were diluted and added to the disposable folded capillary cell. The measurements were done at 25 °C. 

#### 2.3.7. Contact Angle

The contact angle measurement was performed by dropping water on the rubber film using a microsyringe. The water droplet on the sample surface was recorded by a digital microscope. The angle formed between the liquid-solid interface was measured by ImageJ software. 

#### 2.3.8. Differential Scanning Calorimetry

The thermal property of the samples was investigated by differential scanning calorimetry (DSC) using Q2000 (TA Instruments, New Castle, DE, USA). An amount of 5–10 mg of the sample was added into an aluminum pan. The heating rate was 10 °C/min under nitrogen from −80 °C to 200 °C.

#### 2.3.9. Thermogravimetric Analysis

The thermogravimetric analysis was conducted using a TGA/DSC1 (Mettler Toledo, Columbus, OH, USA). An amount of 10 mg of dried sample was added to a sample pan and then inserted into the furnace. The test was carried out in the temperature range of 50 to 600 °C at a heating rate of 10 °C/min under a nitrogen atmosphere. The change in the remaining weight of the sample was recorded continuously during measurement. 

### 2.4. Preparation of Silica-Filled (PAA-co-PAM)-DPNR Composites

The silica-filled natural rubber composites were prepared by using a two-roll mill. The compounding ingredients and the sequence of mixing are shown in Table 2. The DPNR, P10-DPNR, and P30-DPNR were compounded with a sulfur vulcanization system. Various contents of the silica were subjected to a rubber matrix for the preparation of the silica-based natural rubber composites. The silica contents were varied as 10 and 20 phr. 

### 2.5. Characterization of the Silica-Filled (PAA-co-PAM)-DPNR Composites

#### 2.5.1. Cure Characteristics

The rubber compounds were stored for 24 h and tested with a moving die rheometer (MDR) using MDR 3000 (MonTech, Taipei, Taiwan) to determine the optimal time and temperature for vulcanization. The compounds were then cured in a compression molding machine (LabTech engineering company Ltd., Samut Prakan, Thailand). 

#### 2.5.2. Swelling Ratio

The dried samples (1.0 cm × 1.0 cm × 0.2 cm) were immersed in toluene for 24 h. Then, the samples were collected and weighed after wiping them with filter paper. The swelling ratio was calculated using the equation here.

(4) $$\mathrm{Swelling}\;\mathrm{ratio}\;=\frac{\mathrm{Ws}-\mathrm{Wo}}{\mathrm{Wo}}$$

where Ws is the weight of the swollen sample, and Wo is the weight of the dried sample.

#### 2.5.3. Morphology 

The morphology of the silica-filled composites was examined by scanning electron microscope (SEM) using an FEI Quanta 450 (Philips, Hillsboro, OR, USA). The rectangular rubber sheets (3.0 cm × 1.0 cm × 0.2 cm), after vulcanization, were frozen in liquid nitrogen and broken to investigate the dispersion of the silica in the natural rubber matrix [29]. The cross-section of samples was observed by SEM, and the dispersion of the silica was studied by energy dispersive spectroscopy coupled with SEM (SEM/EDS). Moreover, the fracture surface of the samples, after tensile testing, was also examined. The samples were sputter-coated with gold under a vacuum by using a sputter coater before observation with the SEM.

#### 2.5.4. Mechanical Properties

The specimens for tensile measurement were prepared by cutting them into a dumbbell shape according to ASTM D412 [30]. For tear measurement, the specimens were followed by ASTM D624 [31]. The measurement was performed using the universal testing machine 5569 (Instron, Norwood, MA, USA) under the condition of crosshead speed of 500 mm/min with a 10 kN load cell. The measurement was repeated six times for each sample. The ability of the samples to resist deformation by hardness was also determined. The hardness measurement was carried out by HPE Shore A durometer (Bareiss, Oberdischingen, Germany). The samples were measured according to ASTM D2240 [32]. A steel pin was pressed into the rubber surface. The hardness value was recorded. 

#### 2.5.5. Dynamic Mechanical Property

The thermomechanical property of the samples was evaluated via dynamic mechanical analysis using an Eplexor QC 100 N (Gabo, Ahlden, Germany) in the tension mode. The temperature sweep test was carried out with a static strain of 1%, a dynamic strain of 0.1 %, a frequency of 5 Hz, and a temperature in the range of −80 to 100 °C. The heating rate was 2 °C/min [33]. 

## 3. Results and Discussion

### 3.1. Modification of Deproteinized Natural Rubber by Graft Copolymerization with Comonomer of Acrylic and Acrylamide

**Figure 1:**
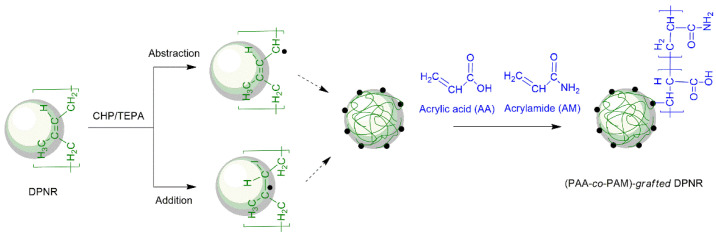
Schematic representation of preparing the (PAA-*co*-PAM)-*grafted* DPNR by graft copolymerization.

The (PAA-*co*-PAM)-*grafted* deproteinized natural rubber ((PAA-*co*-PAM)-DPNR) was prepared by graft copolymerization in the latex stage. The formation of the (PAA-*co*-PAM)-DPNR is shown in Figure 1. The graft copolymerization was initiated by CHP/TEPA redox initiator to generate free radicals on the natural rubber chains at the particle/water interface through an abstraction and addition reaction [34,35]. The grafting occurred at the particle surface of the NR. The comonomer was attached at the grafting sites to produce (PAA-*co*-PAM)-*grafted* DPNR. The effect of the polymerization parameters was investigated in order to find the optimal conditions for the preparation of modified natural rubber.

#### 3.1.1. Effect of Reaction Temperature

**Figure 2:**
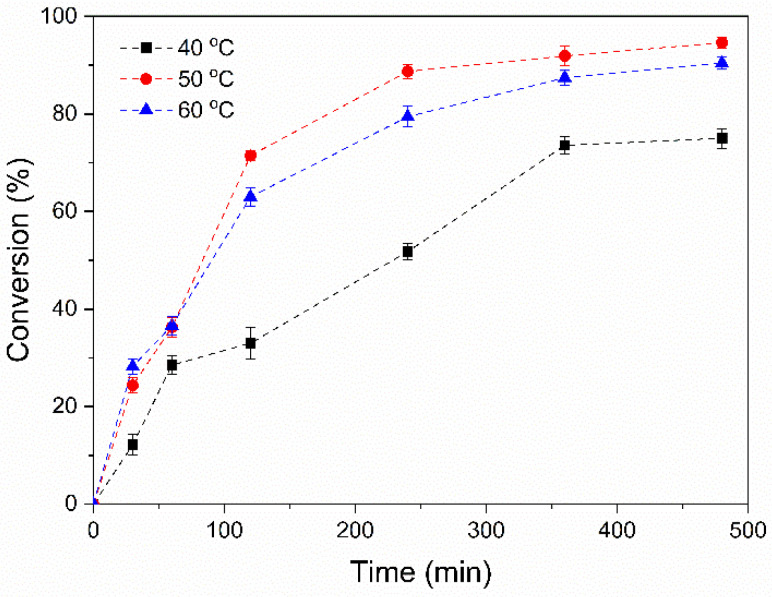
Effect of temperature on the monomer conversion of (PAA-*co*-PAM)-DPNR.

The effect of the reaction temperature on the preparation of the modified DPNR particles was studied. The experiment was performed by using 10 phr of monomer content and 1 phr of the initiator. Figure 2 presents a plot of the monomer conversion as a function of polymerization time at different reaction temperatures (40, 50, and 60 °C). It can be seen that the conversion increased with increasing polymerization time for all temperatures used. The polymerization rate increased rapidly at the initial polymerization stage and remained constant after 360 min. When the reaction temperature increased from 40 to 50 °C, the conversion increased from 73.6 to 91.9%. The increase in temperature can cause a higher collision frequency between monomer molecules and the free radicals in the system. This resulted in an acceleration of the polymerization rate and an increase in monomer conversion [36]. However, after increasing the temperature to 60 °C, the monomer conversion slightly increased at the early stage of polymerization, and then the conversion seemed to decrease when compared to 50 °C. The monomer conversion was reached at 87.4%. Thus, the reaction temperature at 50 °C, which exhibited the highest conversion, was selected for further study.

#### 3.1.2. Effect of Initiator Content

**Figure 3:**
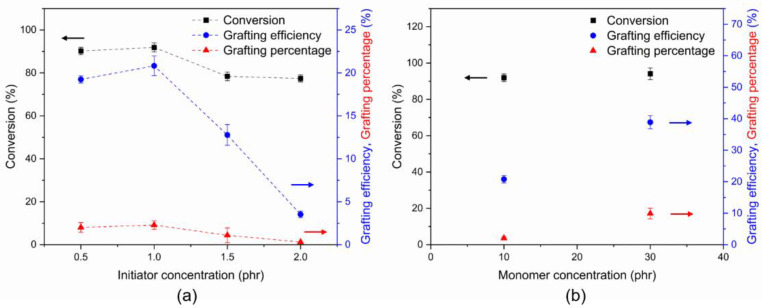
(**a**) Effect of initiator contents; (**b**) effect of monomer contents on the monomer conversion, grafting efficiency and grafting percentage.

The influence of the initiator concentration on conversion, grafting efficiency, and grafting percentage was also investigated. The concentration of the initiator varied within the range of 0.5 to 2.0 phr. From Figure 3a, the monomer conversion was found to be 90.2% for 0.5 phr of the initiator used. When the concentration of the initiator increased to 1.0 phr, the monomer conversion slightly increased to 91.9%. After that, a reduction in monomer conversion was observed when the initiator concentration was increased. The conversion values were decreased to 78.4 and 77.4% for the initiator concentrations of 1.5 and 2.0 phr, respectively. The reduction in monomer conversion occurs because more radicals are formed at higher initiator concentrations and can enhance the probability of chain termination [37,38]. It was also observed that the grafting efficiency and grafting percentage showed the same tendency as the conversion result. At 1.0 phr of initiator, the grafting efficiency and grafting percentage were 20.83 and 2.08, respectively. According to the reaction mechanism, the hydrophobic natural rubber chains were coiled to form particles dispersed in the aqueous phase. The free radicals on the natural rubber chains were generated by the redox initiator, and the vinyl monomers were polymerized to form the grafted product. The grafting can take place on the surface of the natural rubber particles [39]. There was a greater grafting site on the natural rubber chain when the initiator was increased, resulting in an increase in grafting efficiency and grafting percentage. However, the grafting efficiency and grafting percentage decreased when the initiator concentration was 1.5 and 2.0 phr. This is because the vinyl monomers can spontaneously polymerize without grafting to form a homopolymer [40]. Thus, the grafting efficiency and grafting percentage decreased with the excess of the initiator.

#### 3.1.3. Effect of Monomer Content 

Then, the (PAA-*co*-PAM)-DPNR was prepared by varying the monomer contents from 10 phr to 30 phr, which was denoted as P10-DPNR and P30-DPNR, respectively. The effect of monomer concentration was examined using a fixed concentration of initiator (1.0 phr) at a reaction temperature of 50 °C. The resulting product became a more viscous latex when the monomer contents increased due to a greater amount of PAA-*co*-PAM forming in the system. From Figure 3b, the monomer conversion was observed to be around 91.9–94.1% for the monomer concentration of 10–30 phr. The grafting efficiency and grafting percentage were found to increase with the increasing monomer content. The grafting efficiency and grafting percentage were in the range of 20.8–38.9% and 2.1–9.9%, respectively. The modified DPNR was also prepared by graft copolymerization with only acrylic acid (PAA-DPNR) and acrylamide (PAM-DPNR) for comparison. Unstable latex was obtained for the PAA-DPNR. The phase separation of PAA-DPNR leads to the occurrence of a creaming phenomenon. Figure S1 displays the creaming index with different storage times (see Supplementary information), which can indicate the degree of latex stability. The creaming index of PAA-DPNR increased with storage time. Although PAA had the ability to stabilize particles through electrosteric stabilization [41], high acidity in the system could deteriorate the colloidal stability of the rubber particles in an aqueous medium. When the comonomer was used at the ratio of 50:50 acrylic acid/acrylamide by weight percentage, the (PAA-*co*-PAM)-DPNR exhibited good colloidal stability over a month, as was the same for the PAM-DPNR. However, the PAM-DPNR had a low grafting percentage (6.4%) compared to the (PAA-*co*-PAM)-DPNR. The stronger interactions between the copolymers of the polyacrylic acid and polyacrylamide can lead to the formation of self-assembled structures and generate additional physical crosslinking through hydrogen bonding [42]. Therefore, the use of comonomer would be effective for preparing colloidally stable rubber particles with high grafting percentage as compared to a sole monomer.

### 3.2. Characterization of (PAA-co-PAM)-Modified Deproteinized Natural Rubber 

#### 3.2.1. Chemical Structure of the (PAA-*co*-PAM)-Modified DPNR

**Figure 4:**
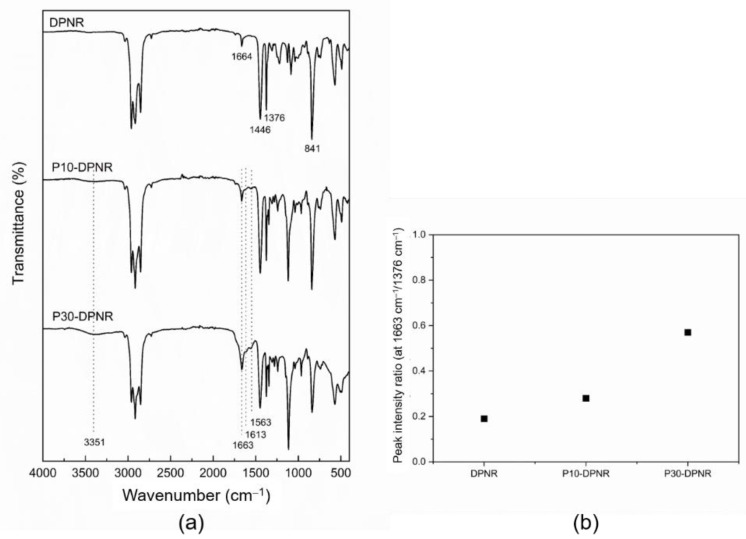
(**a**) FTIR spectra of the (PAA-*co*-PAM)-DPNR prepared by using 10 and 30 phr monomer content after extraction; (**b**) the relationship of peak intensity ratio at 1663 cm^−1^/1376 cm^−1^ of the prepared (PAA-*co*-PAM)-DPNR compared to the DPNR (1664 cm^−1^/1376 cm^−1^).

The chemical structure and functional groups of DPNR and (PAA-*co*-PAM)-modified DPNR were examined by FTIR to confirm graft copolymerization. Figure 4a shows the FTIR spectra of the (PAA-*co*-PAM)-modified DPNR with different monomer contents after extraction to separate the ungrafted PAA-*co*-PAM and compare it to the original DPNR. The FTIR spectrum of the DPNR shows a peak at 1664 cm^−1^, which corresponds to C = C stretching vibration. The peaks at 1446 cm^−1^ and 1376 cm^−1^ are assigned to -CH_2_ and -CH_3_ stretching, respectively. The peak appearing at 841 cm^−1^ corresponds to =CH bending out of plane. These peaks indicate the chemical structure of polyisoprene in natural rubber [43,44]. In the case of (PAA-*co*-PAM)-DPNR at different monomer concentrations after extraction, its spectra showed the important characteristics of natural rubber. Moreover, a broad peak was found at approximately 3500–3200 cm^−1^, indicating NH and OH stretching. The C = O and C-O stretching vibrations appeared at 1663 and 1240 cm^−1^, respectively. The peak at 1563 cm^−1^ was assigned to carboxylate (-COO^−^), obtained by the partial neutralization of the acrylic acid with a basic compound [45]. A peak at 1613 cm^−1^ for NH bending was also observed. These correspond to the functional groups of polyacrylic acid and polyacrylamide [46]. This can indicate the successful grafting of polyacrylic acid and polyacrylamide onto natural rubber. The grafting was also confirmed by solid-state NMR. The CP/MAS ^13^C-NMR spectrum of P30-DPNR (Figure S2, see Supplementary Information) shows characteristic signals both of natural rubber and grafted polymer. The signals at 136.4, 126.9, 34.0, 28.3, and 25.1 ppm were assigned to the isoprene units of natural rubber [47]. Moreover, peaks at around 182.9 ppm (C=O) and 45.5 ppm (CH_2_ and CH) of polyacrylic acid and polyacrylamide also appeared [48]. Furthermore, it was observed that the C = O stretching (1663 cm^−1^) of the (PAA-*co*-PAM)-DPNR from the FTIR spectra was shifted from both PAA-DPNR and PAM-DPNR. As can be seen in Figure S3 (see Supplementary Information), the PAA-DPNR spectrum showed a signal at 1704 cm^−1^ [49], which was attributed to C = O stretching in the carboxylic acid. The signal of C = O in the PAM-DPNR occurred at 1656 cm^−1^ [50]. For the (PAA-*co*-PAM)-DPNR at a comonomer ratio of 50:50 by weight percentage, the C = O shifted to 1663 cm^−1^. These changes indicate the occurrence of hydrogen bonding interactions between polyacrylic and polyacrylamide in the system [51]. Moreover, the peak intensity ratios between the C = O stretching of PAA-*co*-PAM at 1663 cm^−1^ and the -CH_3_ stretching of natural rubber at 1376 cm^−1^ were analyzed, as shown in Figure 4b. From the FTIR spectra, although the peak representing the C = C (1664 cm^−1^) of natural rubber overlapped with the peak of the C = O, the peak intensity ratio of the modified DPNR was higher than that of the DPNR. This demonstrated the presence of grafted PAA-*co*-PAM. The intensity ratios also increased with increasing monomer content. These confirmed the increase in polymer grafted onto the natural rubber, which corresponded to the results calculated from the gravimetric analysis.

#### 3.2.2. Morphology of (PAA-*co*-PAM)-DPNR

**Figure 5:**
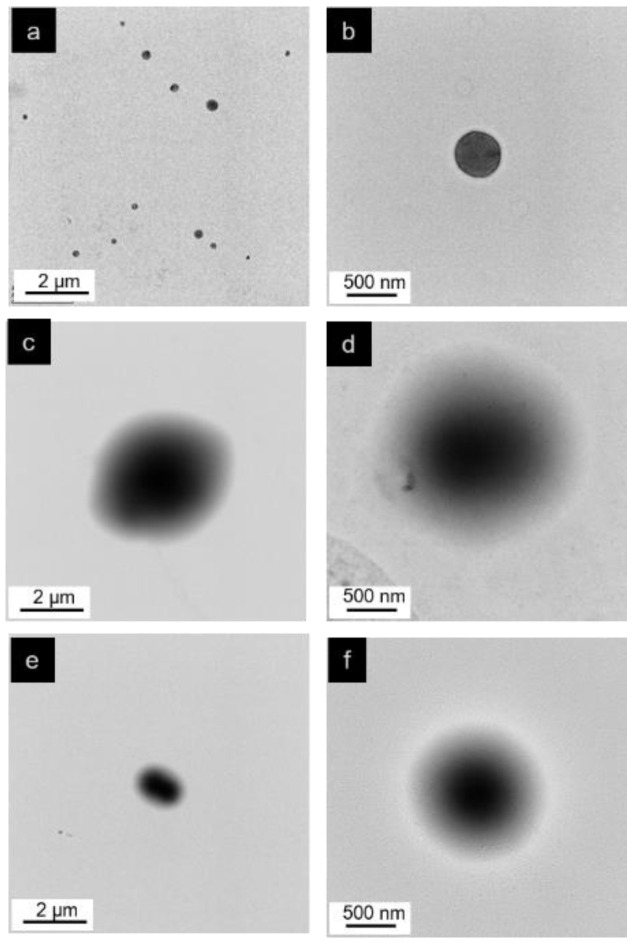
TEM images of (**a**,**b**) DPNR; (**c**,**d**) P10-DPNR; (**e**,**f**) P30-DPNR.

The morphology of the DPNR and the (PAA-*co*-PAM)-modified DPNR particles was studied using a transmission electron microscope, as shown in Figure 5. Their particle size values are summarized in Table 3. The DPNR particles exhibited a dark color and spherical shape, with a size of about 0.384 ± 0.089 µm. After modification, a larger particle size and core shell structure were obtained. It was clearly observed that the rubber particle is the core and is covered with a PAA-*co*-PAM shell [52]. The P10-DPNR and P30-DPNR had a size of about 2.086 ± 0.506 and 0.805 ± 0.222 µm, respectively, which were greater than that of the unmodified DPNR. When the monomer content increased, the size decreased. 

#### 3.2.3. Particle Size and Surface Properties

**Figure 6:**
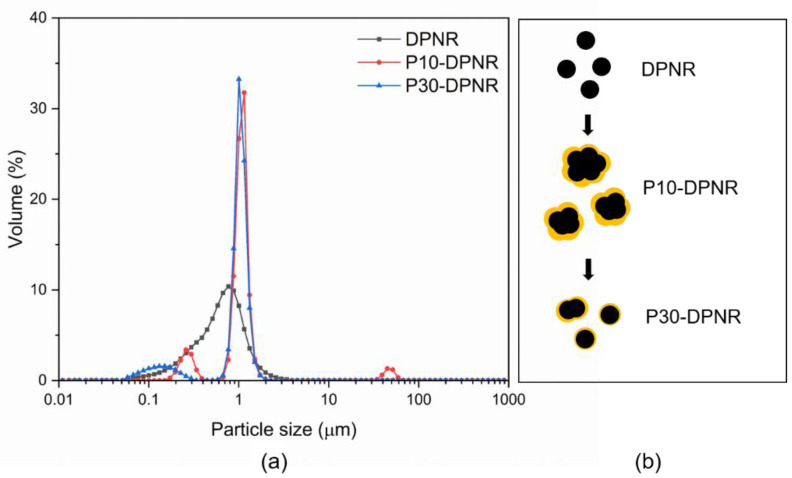
(**a**) Particle size distribution; (**b**) schematic representation of (PAA-*co*-PAM)-covered DPNR.

The particle size distribution and size of the prepared samples were examined using a dynamic light-scattering (DLS) technique, the results of which are displayed in Figure 6a and Table 3, respectively. The DPNR particles had a mean size of 0.610 ± 0.078 µm. After modification by graft copolymerization, the particle size increased. When the monomer was added to 10 phr, the mean particle size was 2.533 ± 0.080 µm. The particle size decreased to 0.874 ± 0.005 µm when a 30 phr monomer was used. The results were consistent with the transmission electron microscopy. A reduction in particle size was found with an increase in monomer content. The increase in monomer content resulted in an increase in the resulting (PAA-*co*-PAM) covering the rubber particles, causing the rubber particles to be separated from each other, as shown in Figure 6b. Moreover, the PAA-*co*-PAM that covered the DPNR particles had the ability to stabilize the rubber particles through electrosteric repulsion [53]. As can be seen from the zeta potential measurements from Table 3, the DPNR exhibited a negative zeta potential (−20 mV). This slightly negative value may be attributed to the remaining proteins in the system. The surface charges of P10-DPNR and P30-DPNR were observed to be −33.2 and −64.5 mV, respectively. The highly negative values might have resulted from the carboxylate groups containing the PAA-*co*-PAM adsorbed onto DPNR particles after modification. They can provide electrosteric repulsion to prevent aggregation and sufficiently stabilize the rubber particles, which are dispersed in the water phase [54].

**Figure 7:**
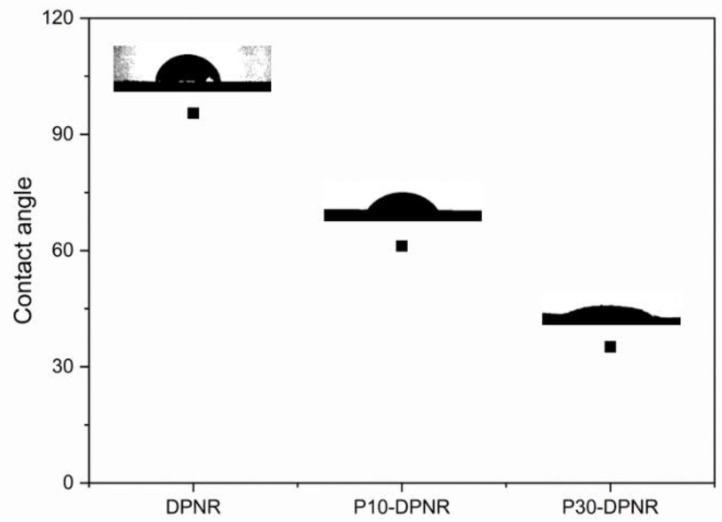
Contact angle of DPNR, P10-DPNR, and P30-DPNR.

Figure 7 shows the contact angle of the DPNR and the (PAA-*co*-PAM)-modified DPNR films. The contact angle was also used to determine the surface properties of the prepared samples. The DPNR film had a contact angle of 95.5°. The contact angle tended to decrease when the grafting percentage of the modified DPNR increased. The P10-DPNR and P30-DPNR had a contact angle of 61.2° and 35.2°, respectively, which decreased by 0.36–0.63 times when compared to the DPNR. The decrease in the contact angle of the modified DPNR indicated that the modified DPNR films were more hydrophilic than the DPNR version [55]. Therefore, grafts using PAA-*co*-PAM can enhance the polarity of natural rubber. This might be useful for preparing composites by mixing them with polar reinforcing agents, such as silica, to improve the mechanical properties of the composites.

#### 3.2.4. Thermal Properties

**Figure 8:**
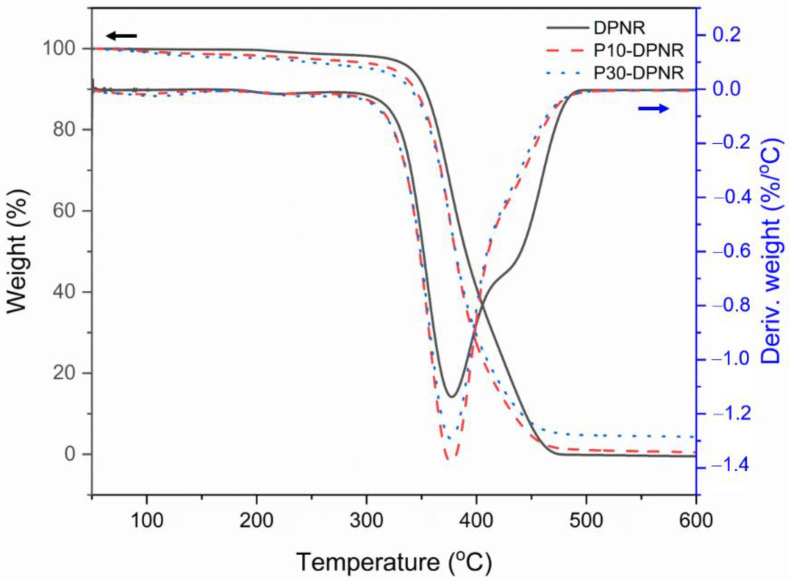
TGA and DTG thermograms of DPNR, P10-DPNR, and P30-DPNR.

The thermal properties of the modified DPNR were investigated by DSC. The glass transition temperature (Tg) determined from the DSC result is summarized in Table 3. The Tg of the DPNR was around −65.0 °C. For the modified DPNR, their Tg values slightly increased with increasing amounts of grafting percentage. The Tg values for P10-DPNR and P30-DPNR were observed to be −64.0 and −62.9 °C, respectively. The coverage of PAA-*co*-PAM on the DPNR by graft copolymerization restricted the rubber chain mobility, resulting in a slight shift in the Tg to a higher temperature [56]. The TGA and DTG thermograms of DPNR, P10-DPNR, and P30-DPNR are presented in Figure 8. It was observed that the DPNR decomposed within the temperature range of 336–476 °C [57]. For the P10-DPNR and P30-DPNR, the weight loss at 70–170 °C was attributed to the loss of water in the modified DPNR samples. A minor decomposition at 170–291 °C due to the breakage of the carboxylic and amide side groups of the grafted polymer was also observed [58]. An increase in weight loss during minor decomposition was observed when the grafting percentage increased. At temperatures from 336–420 °C, major decomposition was observed, which corresponded to the polymer backbone of PAA-*co*-PAM [59] and the DPNR. This confirms the presence of DPNR and PAA-*co*-PAM in the modified samples.

### 3.3. Preparation and Properties of the Silica-Filled (PAA-co-PAM)-DPNR Composites

#### 3.3.1. Cure Characteristics

**Figure 9:**
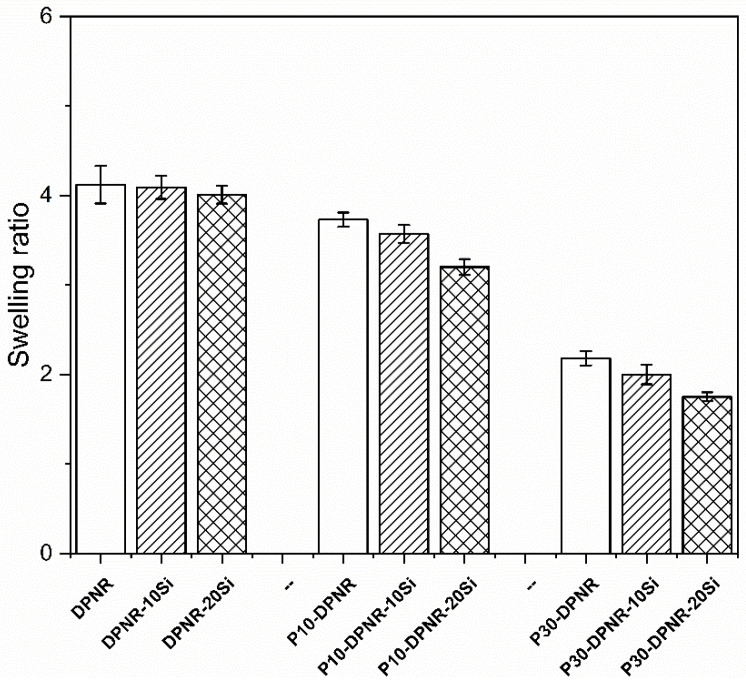
Swelling ratio of the silica-filled natural rubber composites.

**Figure 10:**
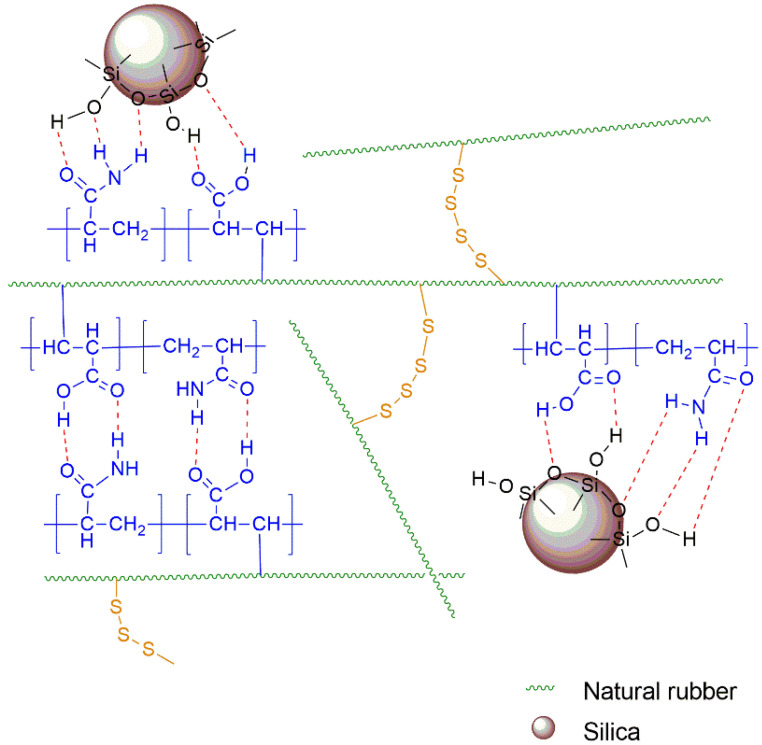
Schematic representation of (PAA-*co*-PAM)-DPNR/silica interactions.

The natural rubber compounds mixed with different concentrations of silica were prepared by using a two-roll mill. The optimal temperature and time for vulcanization were determined by a moving die rheometer (MDR). The cure characteristics of all the compounds obtained from the MDR at a vulcanizing temperature of 150 °C are shown in Table 4. For the compounds without silica, the (PAA-*co*-PAM)-DPNR compounds exhibited a shorter scorch time and longer cure time when compared to the DPNR compound. In this case, the DPNR was functionalized with a copolymer consisting of carboxylic acid and amide groups. These additional groups may affect the vulcanization process. The vulcanization reaction was accelerated by the presence of amine-containing substances, resulting in a decrease in scorch time [60]. On the other hand, vulcanization retardation occurred, which might have increased the vulcanization time, as is seen in the case of existing methacrylic acid in rubber composites [61]. After that, the silica-filled rubber composites were prepared by mixing them with different concentrations of silica at 10 and 20 phr. Generally, silica contributes to the vulcanization system by delaying the rate of vulcanization [62]. Considering the chemical structure of silica, it possesses silanol groups that can adsorb the accelerators in the system, resulting in higher scorch times and cure times [63]. In the case of silica-filled DPNR, it was clearly observed that the scorch time and cure time of DPNR-10Si and DPNR-20Si were higher than that of the DPNR compound without silica, and, consequently, the cure rate index decreased. However, the (PAA-*co*-PAM)-DPNR with 10 and 20 phr of silica showed an increase in the cure rate, with shorter scorch and cure times when compared to silica-filled DPNR using the same amount of silica. It could be that the polar functional groups contained in the modified DPNR play an important role by interacting with silica in the mixing step. Therefore, the adsorption of the accelerators was reduced, resulting in an increase in the cure rate. The presence of (PAA-*co*-PAM) and silica in the rubber compounds revealed important features. At 10 phr of silica loaded into the (PAA-*co*-PAM)-DPNR compounds, a longer scorch time and shorter cure time were observed when compared to the (PAA-*co*-PAM)-DPNR without silica. For the processing, the longer scorch time enhances processing safety, while a shorter cure time is preferred in terms of better productivity [60]. When the silica content was increased to 20 phr, the scorch time and cure time increased for the modified rubber compounds using 10 phr of silica. Although some silica interacted with the modified DPNR, more of the free silica from the higher loadings had the ability to adsorb the accelerators, leading to the retardation of the vulcanization process. In addition, the minimum torque (M_L_) of the (PAA-*co*-PAM)-DPNR was higher than that of the DPNR. It was also found that the M_L_ increased with an increase in silica content in the compounds. The M_L_ is related to the viscosity of the rubber compounds. The increase in M_L_ was probably due to the PAA-*co*-PAM chains and the rigid silica particles restricting the motion of the rubber chains [64]. Moreover, the delta torque (M_H_-M_L_) of the (PAA-*co*-PAM)-DPNR was higher than that of the DPNR, and the addition of silica presented an increase in the delta torque. The delta torque was related to the crosslinking density of the vulcanizates. These results corresponded with the swelling tests of the rubber composites in toluene, as shown in Figure 9. The high crosslinking density of the rubber vulcanizates leads to a reduction in the swelling degree [65]. It was observed that the DPNR had a swelling ratio of 4.12. The swelling ratio decreased to 3.73 and 2.18 for P10-DPNR and P30-DPNR, respectively. At the same time, the swelling ratio tended to decrease with an increase in silica content, which was clearly observed in the case of the silica-filled (PAA-*co*-PAM)-DPNR composites. For example, the swelling ratio values were found to be 3.73, 3.57, and 3.20 for the P10-DPNR mixed with 0, 10, and 20 phr, respectively. The grafting of (PAA-*co*-PAM) onto the DPNR, together with the addition of silica, exhibited a noticeable increase in the delta torque and a decrease in the swelling ratio, indicating the high crosslink density in the structure. Their interactions are illustrated in Figure 10. The increase in crosslink density is probably due to the crosslinking reaction via sulfur vulcanization, together with the hydrogen bonding of the (PAA-*co*-PAM) copolymer chains and the interaction between the grafted polar functional groups and the silica [66]. 

#### 3.3.2. Morphology of Silica-Filled Natural Rubber Composites

**Figure 11:**
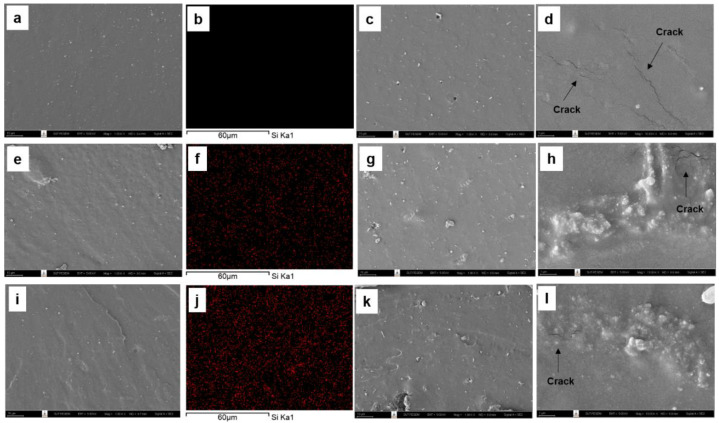
SEM images of freeze fracturing, corresponding EDS (the red spots indicate silica particles), and tensile fracture surface at ×1000 and ×10,000 magnifications of (**a**–**d**) DPNR; (**e**–**h**) DPNR-10Si; (**i**–**l**) DPNR-20Si.

**Figure 12:**
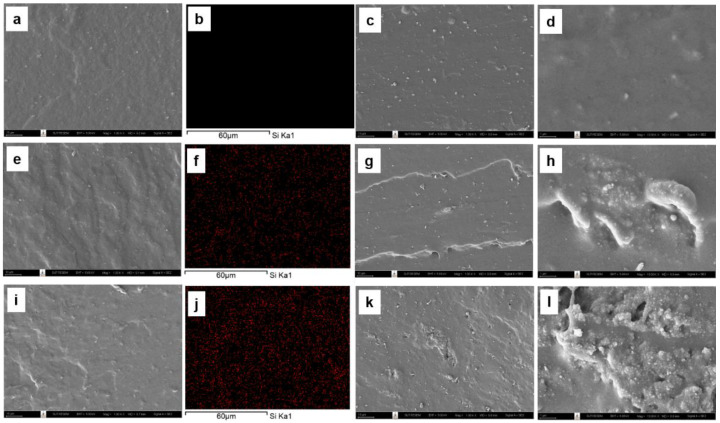
SEM images of freeze fracturing, corresponding EDS (the red spots indicate silica particles), and tensile fracture surface at ×1000 and ×10,000 magnifications of (**a**–**d**) P10-DPNR; (**e**–**h**) P10-DPNR-10Si; (**i**–**l**) P10-DPNR-20Si.

**Figure 13:**
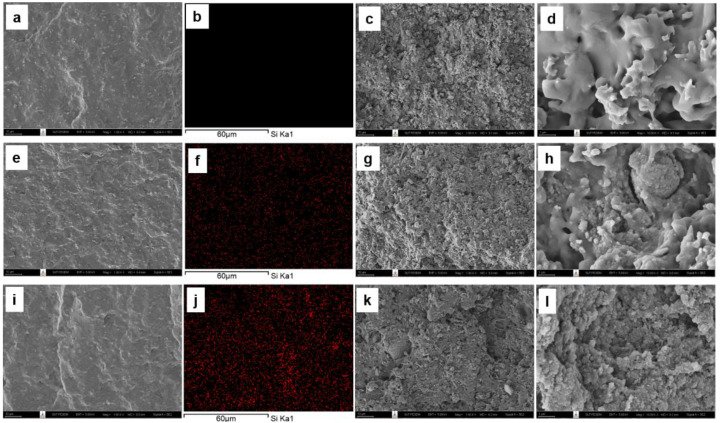
SEM images of freeze fracturing, corresponding EDS (the red spots indicate silica particles), and tensile fracture surface at ×1000 and ×10,000 magnifications of (**a**–**d**) P30-DPNR; (**e**–**h**) P30-DPNR-10Si; (**i**–**l**) P30-DPNR-20Si.

The morphology of the silica-filled DPNR, silica-filled P10-DPNR, and silica-filled P30-DPNR composites, with varying silica content, was examined by scanning electron microscope, as demonstrated in Figure 11, Figure 12 and Figure 13, respectively. The cross-section surface of all vulcanizates (by freeze fracturing with nitrogen liquid) showed a smooth surface with some compounding ingredients appearing on their surfaces. When the silica was added into the system, good distribution of the silica particles in the natural rubber matrix was obtained for the composites with 10 phr of silica, as observed from the SEM/EDS mapping. However, some aggregation of the silica particles was observed under high-loading content (20 phr). The samples were then subjected to tensile tests, and the fracture surface was examined by SEM. The tensile fracture surface of the DPNR was smooth, as shown in Figure 11c,d. We observed holes and small cracks throughout its surface. This is because the compounding ingredients are pulled out from the tensile force, which was attributed to poor adhesion with the natural rubber matrix [67]. For the P10-DPNR in Figure 12c,d and the P30-DPNR in Figure 13c,d, the embedment of the compounding ingredients into the surface was observed. The morphology clearly changed after tensile measuring, leading to a rough surface. The modification of natural rubber by PAA-*co*-PAM grafting strongly affects its fracture morphology, indicating the rigid structure of the modified rubber [68]. For the DPNR with the addition of silica at 10 phr in Figure 11g,h and 20 phr in Figure 11k,l, cracks in the rubber matrix at the region between the natural rubber and the silica cluster were found due to the weak interaction and incompatibility of both of the components. In the case of the silica-loaded (PAA-*co*-PAM)-DPNR samples, uneven microscopic layers and rough surfaces were observed. The SEM images in Figure 12 and Figure 13g,h,k,l clearly revealed the extended region of the NR matrix-silica clusters after deformation [69]. Therefore, the grafting of DPNR with polar components might improve the interaction between natural rubber and fillers. This overall remarkable morphology change was due to the strong interfacial adhesion between the modified DPNR and the silica.

#### 3.3.3. Mechanical Properties of Composites

**Figure 14:**
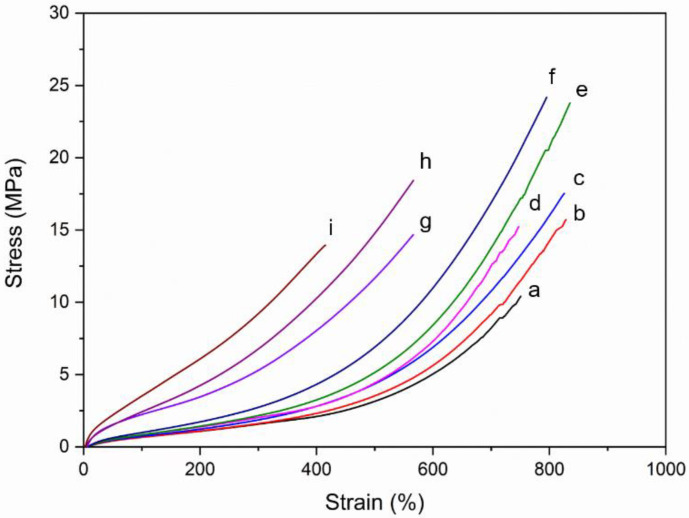
Stress-strain curve of the (**a**) DPNR; (**b**) DPNR-10Si; (**c**) DPNR-20Si; (**d**) P10-DPNR; (**e**) P10-DPNR-10Si; (**f**) P10-DPNR-20Si; (**g**) P30-DPNR; (**h**) P30-DPNR-10Si; (**i**) P30-DPNR-20Si composites.

**Figure 15:**
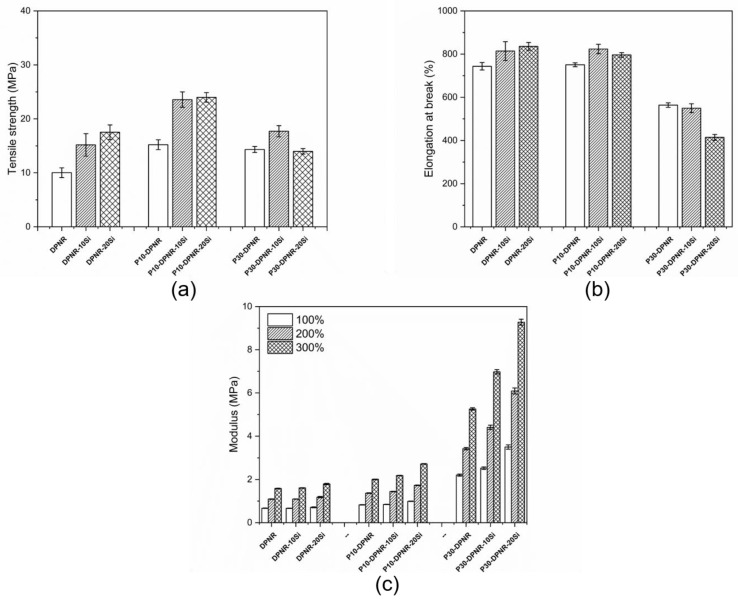
(**a**) Tensile strength; (**b**) elongation at break; and (**c**) modulus at 100, 200, and 300% strain of the silica-filled natural rubber composites.

Figure 14 displays the stress-strain curve obtained from the tensile testing of the DPNR and (PAA-*co*-PAM)-DPNR mixed with different silica contents. It was observed that the stress gradually increased after deformation. After that, the stress sharply increased. This is because of the occurrence of the strain-induced crystallization of the natural rubber chains during deformation. The sample was tested under tension mode until it broke. From the results, comparisons between the tensile strength, elongation at break, and modulus at 100, 200, and 300% strain are shown in Figure 15a–c, respectively. The P10-DPNR and P30-DPNR exhibited good mechanical properties by showing an enhanced tensile strength and modulus compared to the DPNR. The tensile strength of P10-DPNR and P30-DPNR were 15.21 and 14.33 MPa, respectively. The DPNR had a tensile strength of 10.02 MPa. At the same time, the modulus values, under a 300% strain, for P10-DPNR and P30-DPNR were found to be 2.01 and 5.26 MPa, respectively, which were higher than that of the DPNR (1.58 MPa). The elongation at break of P10-DPNR (750.75%) was not much different from that of the DPNR (743.58%). When the grafting percentage was increased, as was the case for P30-DPNR, its elongation at break was reduced to 563.91%. This was probably because the polyacrylic acid (the Tg of PAA was 100–125 °C) [70,71] and polyacrylamide (Tg of PAM was 190 °C) [72] became more rigid and restricted the movement of the chains in the natural rubber during deformation. For the silica-filled composites, an increase in tensile strength and modulus was obtained. This indicates that silica acts as a reinforcing filler in the rubber matrix and can improve the mechanical properties of the composites. For example, the tensile strength of P10-DPNR-10Si increased by 1.55 and 2.35 times when compared to P10-DPNR and DPNR, respectively. Moreover, the tensile strength of P10-DPNR-10Si increased by 1.55 times when compared to DPNR-10Si, suggesting strong interactions between the silica and natural rubber after modification. It was noticed that the tensile strength of those composites with 10 phr of silica significantly increased when compared to those samples without silica. However, the tensile strength of P10-DPNR-20Si saw no difference when compared to P10-DPNR-10Si. The addition of 10 phr of silica did not affect the elongation at break of the composite. In addition, at 20 phr of silica, some properties were reduced due to the aggregation of the silica particles at high content loading. Therefore, the addition of 10 phr silica would be suitable for the reinforced composites.

**Figure 16:**
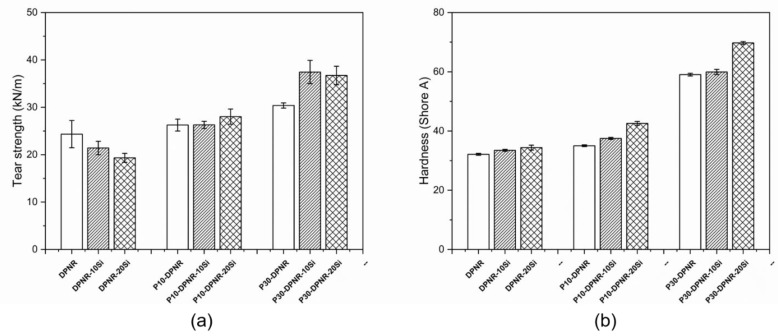
(**a**) Tear strength and (**b**) hardness of the silica-filled natural rubber composites.

Figure 16a displays the tear strength of those composites prepared from DPNR and (PAA-*co*-PAM)-DPNR, with the addition of various contents of silica. It was observed that the tear strength of the silica-filled DPNR composites seemed to decrease with an increase in silica content. Natural rubber molecules are composed of isoprene units, which are nonpolar. Therefore, a weaker natural rubber/silica interaction was obtained [73]. However, the P10-DPNR and P30-DPNR showed an increase in tear strength when compared to the DPNR. Their tear strength values increased when the silica was added. This was suggested by the strong networks being formed in the composites and the strong interactions between the modified natural rubber and silica. Moreover, the ability of the materials to resist permanent deformation via a compression load on the sample surface was measured via hardness testing, as shown in Figure 16b. The hardness of the DPNR was observed to be 32.1. The (PAA-*co*-PAM)-DPNR was harder than the DPNR. The hardness values of P10-DPNR and P30-DPNR increased to 35.0 and 59.0, respectively. This indicated that the (PAA-*co*-PAM)-DPNR had a higher rigidity and resistance performance to the deformation. In addition, the hardness of the composites appeared to increase with an increase in silica content. Silica particles are rigid fillers and have good dispersion in the rubber matrix, as can be seen from the SEM/EDS results. Thus, the hardness of the silica-filled composites was enhanced. From these results, the modification of natural rubber and the incorporation of silica could improve the mechanical properties of these composites. The modification of natural rubber increased its polarity and promoted compatibility with silica after compounding, resulting in a greater reinforcement performance [74].

#### 3.3.4. Dynamic Mechanical Analysis 

**Figure 17:**
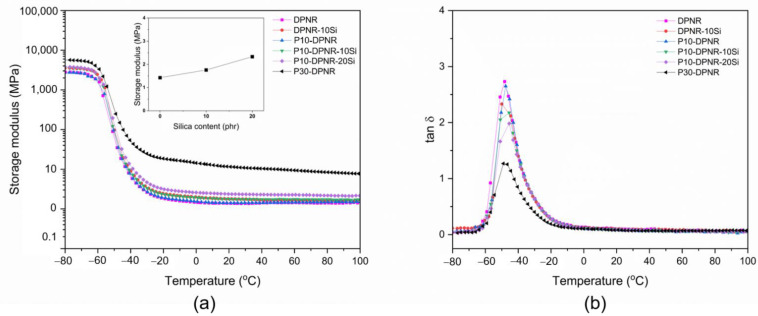
(**a**) Storage modulus: inset; storage modulus of silica-filled P10-DPNR as a function of silica content; (**b**) loss tangent (tan δ) of silica-filled natural rubber composites as a function of temperature.

The thermomechanical behavior of the silica-filled natural rubber composites was studied by temperature sweep testing. Figure 17a,b display the storage modulus (E’) and loss tangent (tan δ) of the silica-filled natural rubber composites as a function of temperature from −80 to 100 °C, respectively. From Figure 15a, all samples had a high E’ value in the glassy state at a low temperature. The E’ decreased when a higher temperature was applied due to the increase of mobility of the chain segments. The E’ dramatically decreased when the temperature was in the range of −50 to −10 °C, which corresponds to the transition region. Then, when the temperature increased from −10 to 100 °C, the E’ did not change much within this range. From the results, the 10-DPNR and P30-DPNR vulcanizates exhibited a higher E’ compared to the DPNR for all temperatures used. The increase in E’ was related to the amount of grafting percentage. For example, at a temperature of 30 °C, the E’ of the DPNR vulcanizate was 1.36 MPa, while the E’ values of the P10-DPNR and P30-DPNR vulcanizates increased to 1.42 and 10.81 MPa, respectively. This was attributed to their higher stiffness after modification. When studying the effect of silica in the composites, the P10-DPNR with various contents of silica (10 and 20 phr) was compared to P10-DPNR without the addition of silica, as shown in Figure 15a (inset). The E’ of the silica-filled P10-DPNR was higher than that of the P10-DPNR and tended to increase with increasing silica content due to the stronger modified rubber–filler interaction [75], suggesting that more energy was required for its deformation. In addition, the Tg values can be estimated from the tan δ at the transition region, as shown in Figure 15b and Table 5. It was found that the Tg values for P10-DPNR and P30-DPNR shifted to a higher temperature when compared to the DPNR. It was also observed that the shift in Tg for the silica-loaded P10-DPNR was higher (temperature) than for the unloaded silica sample. The Tg of DPNR-10Si shifted to a lower temperature. This may be due to the weak interaction between the DPNR and silica [76]. The Tan δ is a crucial parameter to determine the viscoelastic properties of the materials, which is the ratio of the loss modulus to the storage modulus. From Table 5, the highest tan δ peak was found for DPNR, which exhibited a more viscous response due to having the highest chain mobility. A reduction in the tan δ peak height was observed after grafting with (PAA-*co*-PAM) and/or the addition of the silica particles. For the same amount of silica, the tan δ peak height for P10-DPNR-10Si was lower than that of DPNR-10Si. This could indicate that the restriction of rubber chain movement (via the grafting with PAA-*co*-PAM) and the strong interaction between the modified rubber and silica demonstrated a change in the Tg and tan δ peak [77]. This resulted in an enhanced elastic behavior in the composites. Therefore, the (PAA-*co*-PAM)-modified DPNR and the incorporation of silica exhibited a higher storage modulus and lower tan δ peak, indicating higher stability to the deformation when compared to the DPNR. 

## 4. Conclusions

(PAA-*co*-PAM)-*grafted* deproteinized natural rubber particles were successfully prepared through emulsion graft copolymerization in the latex stage. The optimum conditions were a reaction time of 360 min, a reaction temperature of 50 °C, and an initiator concentration of 1.0 phr. Monomer conversion was found in the range of 91.9–94.1%. The percentage of grating efficiency and grafting percentage were approximately 20.8–38.9% and 2.1–9.9%, respectively. The PAA-*co*-PAM covered the DPNR surface, as observed by TEM, bringing colloidal stability to the DPNR particles via electrosteric stabilization. The modification of the DPNR can enhance hydrophilicity, which was investigated by water contact angle measurement. The contact angle of the natural rubber decreased from 95.5° in the DPNR to 35.2° in the P30-DPNR, indicating enhanced polarity. It is worth mentioning that increasing polarity may improve the compatibility between the silica and rubber, which, consequently, improves the tensile strength, tear strength, and hardness. Moreover, the swelling of the silica-filled (PAA-*co*-PAM)-DPNR in a nonpolar solvent decreased due to the H-bonding interaction with silica. In addition, the composite made from grafting PAA-*co*-PAM onto the DPNR exhibited an increase in storage modulus and a decrease in tan δ peak, indicating that the silica-filled (PAA-*co*-PAM)-DPNR composites had more thermal stability when compared to the DPNR. The modified natural rubber composites in this study showed superior mechanical properties, which can be employed in many rubber applications.

## Figures and Tables

**Table 1:** Preparation of (PAA-*co*-PAM)-modified DPNR.

Ingredients	Content (phr)	
	P10-DPNR	P30-DPNR
DPNR	100	100
Terric16A	5	5
CHP	1	1
TEPA	1	1
Acrylic acid	5	15
Acrylamide	5	15

**Table 2:** Formulations of the silica-filled (PAA-*co*-PAM)-DPNR composites.

Samples	Content (phr)								
	DPNR	DPNR- 10Si	DPNR- 20Si	P10-DPNR	P10-DPNR-10Si	P10-DPNR-20Si	P30-DPNR	P30-DPNR-10Si	P30-DPNR- 20Si
DPNR or modified DPNR	100	100	100	100	100	100	100	100	100
Silica	0	10	20	0	10	20	0	10	20
ZnO	5	5	5	5	5	5	5	5	5
Steric acid	2	2	2	2	2	2	2	2	2
CBS	1	1	1	1	1	1	1	1	1
DPG	1	1	1	1	1	1	1	1	1
Sulfur	1.5	1.5	1.5	1.5	1.5	1.5	1.5	1.5	1.5

**Table 3:** Particle size, zeta potential, and Tg of DPNR and (PAA-*co*-PAM)-DPNR with different monomer contents.

No	Samples	Monomer Content (phr)	Particle Size (µm)		Zeta Potential (mV)	Tg ^2^ (°C)
			SEM ^1^	DLS		
1	DPNR	0	0.384 ± 0.089	0.610 ± 0.078	−20.1	−65.0
2	P10-DPNR	10	2.086 ± 0.506	2.533 ± 0.080	−33.2	−64.0
3	P30-DPNR	30	0.805 ± 0.222	0.874 ± 0.005	−64.5	−62.9

^1^ Measured by imageJ software. ^2^ Determined by DSC.

**Table 4:** Cure parameters obtained from MDR at a vulcanizing temperature of 150 °C.

No	Samples	Natural Rubber (phr)	Silica (phr)	T_s2_ (min)	T_c90_ (min)	ML (dNm)	MH (dNm)	MH-ML (dNm)	CRI (min^−1^)
1	DPNR	100	0	1.55	3.71	0.20	8.71	8.51	46.29
2	DPNR-10Si	100	10	2.50	6.22	0.30	8.82	8.52	26.88
3	DPNR-20Si	100	20	3.24	6.45	0.36	9.57	9.21	31.15
4	P10-DPNR	100	0	0.91	5.35	0.50	9.99	9.49	22.52
5	P10-DPNR-10Si	100	10	1.61	3.62	0.50	11.05	10.55	49.75
6	P10-DPNR-20Si	100	20	2.02	3.94	0.60	12.78	12.18	52.08
7	P30-DPNR	100	0	1.16	4.05	0.50	10.95	10.45	34.60
8	P30-DPNR-10Si	100	10	1.33	3.26	0.55	12.51	11.96	51.81
9	P30-DPNR-20Si	100	20	1.35	4.67	1.37	19.16	17.79	30.12

**Table 5:** Viscoelastic properties of vulcanizates.

Samples	DPNR	DPNR-10Si	P10-DPNR	P10-DPNR-10Si	P10-DPNR-20Si	P30-DPNR
Tg (°C)	−48.06	−49.98	−47.87	−45.47	−45.49	−47.60
Tan δ peak height	2.73	2.33	2.65	2.18	1.98	1.26

## Data Availability

Not applicable.

## Supplementary Materials

The following supporting information can be downloaded at: https://www.mdpi.com/article/10.3390/polym14214602/s1, Figure S1: Creaming index of (PAA-*co*-PAM)-DPNR prepared by using weight ratio of acrylic acid and acrylamide at 50:50 compared to PAA-DPNR and PAM-DPNR; Figure S2: Solid ^13^C-NMR spectrum of P30-DPNR; Figure S3; FTIR of (PAA-*co*-PAM)-DPNR at different weight ratio of acrylic acid and acrylamide.
