# Supplementary material for: Preparation of Poly(acrylic acid-co-acrylamide)-Grafted Deproteinized Natural Rubber and Its Effect on the Properties of Natural Rubber/Silica Composites
Source: Polymers (Basel). 2022 Oct 29;14(21):4602. doi: 10.3390/polym14214602 (PMC9657069; doi:10.3390/polym14214602)
Supplement: Supplementary file 1 [file polymers-14-04602-s001.zip › polymers-1994607-supplementary.pdf]

Supplementary Information

Preparation of Poly(acrylic acid-*co*-acrylamide)-*grafted* Deproteinized Natural Rubber and Its Effect on the Properties of Natural Rubber/silica Composites

Supharat Inphonlek [1,2], Namthip Bureewong [1,2], Kasama Jarukumjorn [1,2], Pranee Chumsamrong [1,2], Chaiwat Ruksakulpiwat [1,2,*] and Yupaporn Ruksakulpiwat [1,2,*]

[1] School of Polymer Engineering, Institute of Engineering, Suranaree University of Technology, Nakhon Ratchasima 30000, Thailand

[2] Research Center for Biocomposite Materials for Medical Industry and Agricultural and Food Industry, Suranaree University of Technology, Nakhon Ratchasima 30000, Thailand

* Correspondence: charuk@sut.ac.th (C.R.); yupa@sut.ac.th (Y.R.)

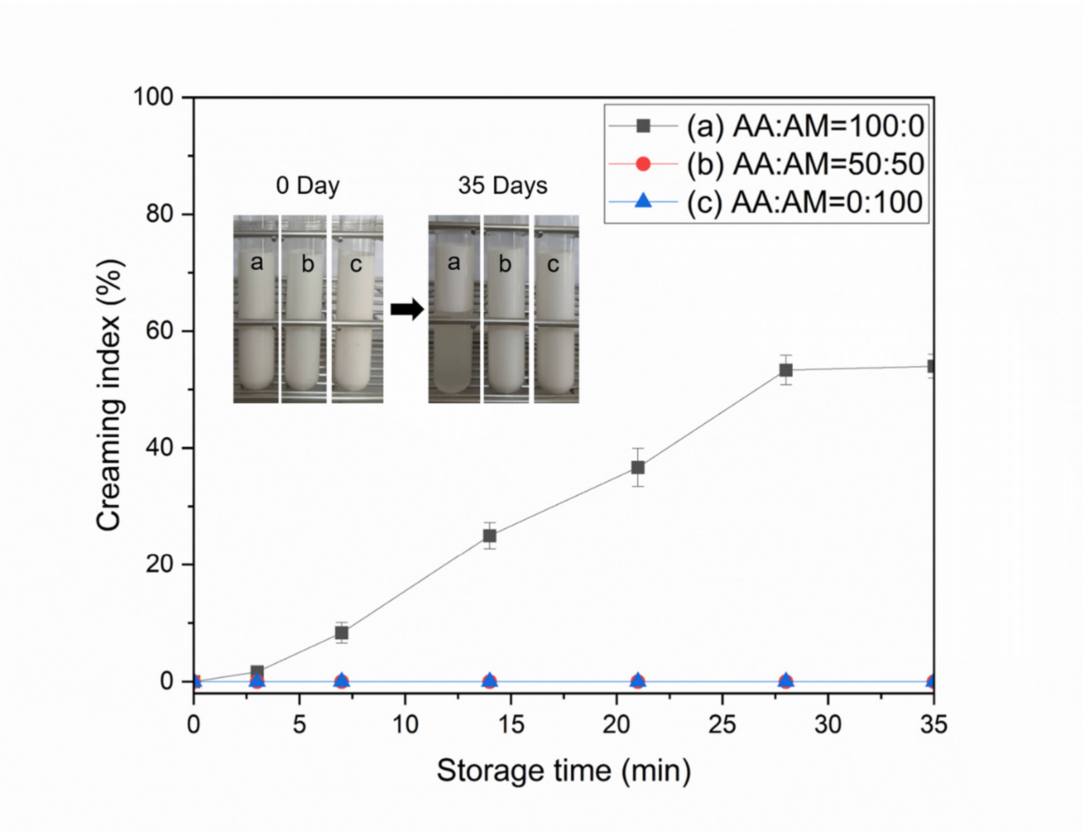

Figure S1. Creaming index of (PAA-*co*-PAM)-DPNR prepared by using weight ratio of acrylic acid and acrylamide at 50:50 compared to PAA-DPNR and PAM-DPNR.

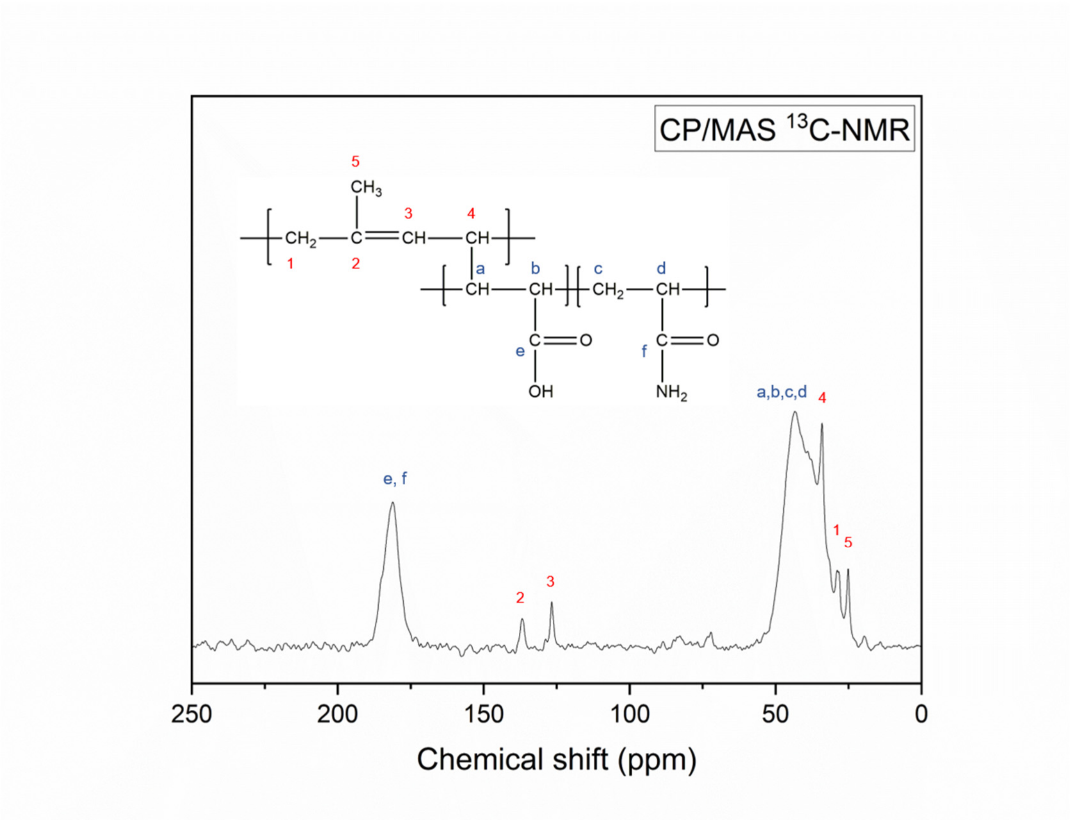

Figure S2. Solid ^{13}C-NMR spectrum of P30-DPNR.

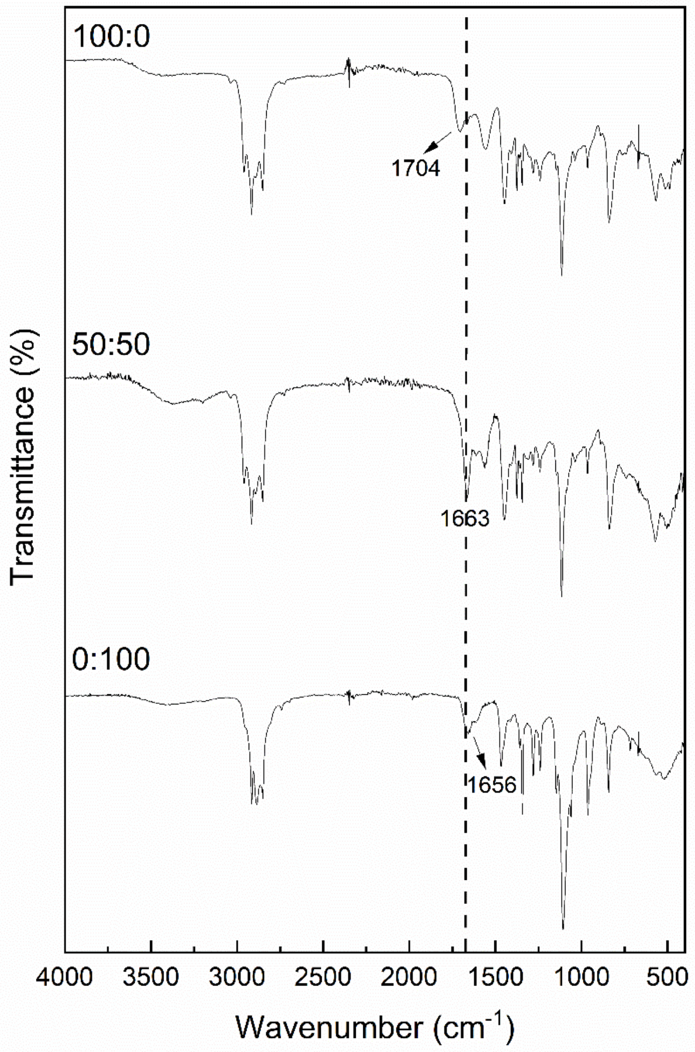

Figure S3. FTIR of (PAA-*co*-PAM)-DPNR at different weight ratio of acrylic acid and acrylamide.
